# Adipocyte-derived Periostin mediates glucocorticoid-induced hepatosteatosis in mice

**DOI:** 10.1016/j.molmet.2019.11.003

**Published:** 2019-11-09

**Authors:** Jian Wan, Yi Shan, Xi Song, Song Chen, Xinyuan Lu, Jie Jin, Qing Su, Bin Liu, Wanju Sun, Bo Li

**Affiliations:** 1 Department of Emergency and Critical Care Medicine, Shanghai Pudong New Area People's Hospital, Shanghai University of Medicine and Health Sciences, Shanghai 201299, China; 2 Department of Emergency and ICU, Changzheng Hospital, Second Military Medical University, Shanghai 200003, China; 3 Department of Endocrinology, XinHua Hospital, Shanghai Jiao Tong University School of Medicine, Shanghai 200092, China; 4 Hubei Key Laboratory for Kidney Disease Pathogenesis and Intervention, Hubei Polytechnic University School of Medicine, Huangshi, Hubei 435003, China

**Keywords:** Periostin, Glucocorticoid, Fatty liver, Adipocyte, Hyperglycemia

## Abstract

**Objective:**

Long-term glucocorticoids (GCs) therapy usually causes many metabolic side effects, including fatty liver. However, the molecular mechanisms remain poorly understood. Herein, we explored the molecular basis of GCs in the development of fatty liver.

**Methods:**

C57BL/6 male mice were injected with Dexamethasone (DEX) while mouse primary hepatocytes (MPHs), HepG2 and Hep1-6 cells were cultured in the presence of DEX. Genes expression in liver tissues and hepatocytes were assessed by quantitative real-time PCR and western blotting, respectively. To explore whether Periostin is involved in the development of GCs-induced fatty liver, wild-type and Periostin knockout mice were treated with DEX or vehicle control. Luciferase reporter and chromatin immunoprecipitation assays were used to determine the regulatory roles of GCs on Periostin expression.

**Results:**

We show that treatment of dexamethasone (DEX), a synthetic analog of GCs, led to the accumulation of triglycerides in the livers of mice, but not in cultured hepatocytes, suggesting that GCs may promote liver steatosis through integrative organ crosstalk mediated by systemic factors. We further found that DEX upregulated the expression levels of Periostin in white adipose tissues, which in turn promoted liver steatosis. Administration of a Periostin-neutralizing antibody or genetic ablation of Periostin largely attenuated DEX-induced hepatic steatosis in mice.

**Conclusions:**

Our findings provided a novel insight that GCs could promote liver steatosis through integrative organ crosstalk mediated by white fat-secreted Periostin. These results establish Periostin as an endocrine factor with therapeutic potential for the treatment of GCs-associated fatty liver.

## Highlights

• Dexamethasone (DEX) treatment led to triglycerides accumulation in the liver of mice, but not in cultured hepatocytes.
• DEX treatment upregulates Periostin in white adipose tissues, which in turn induces liver steatosis in mice.
• Genetic ablation or pharmacological inhibition of Periostin partially attenuated DEX -induced hepatic steatosis in mice.

## 1 Introduction

Triglycerides (TGs) are usually stored in white adipose tissues as an energy source. However, aberrant TGs accumulation in peripheral tissues, such as the liver, is one aspect of the metabolic syndrome and associated with the development of insulin resistance, type 2 diabetes, atherosclerosis, hypertension, and even coronary heart disease [[1], [2], [3], [4]]. It can also trigger a progressive cascade of liver diseases, including steatohepatitis, fibrosis, cirrhosis and hepatocellular carcinoma [5]. Steatosis occurs when the rate of hepatic fatty acid uptake from plasma and de novo fatty acid synthesis are greater than the rate of fatty acid oxidation and export [2,6]. Besides, recent studies suggest that hepatic steatosis is a combination state of dysfunction of other metabolic organs (adipose tissues and skeletal muscles) and dysregulation of hepatic TGs homeostasis. For instance, leptin, adiponectin and Neuregulin 4, which are abundantly expressed in adipose tissues, have been implicated as critical endocrine checkpoints for the development of liver steatosis and steatohepatitis [[7], [8], [9], [10]].

Glucocorticoids (GCs) are widely used in the treatment of acute inflammatory, allergic, immunologic, and malignant disorders. However, long-term GCs therapy is associated with many metabolic side effects, including hyperglycemia, hypertension, and hepatic steatosis [11,12]. At the cellular level, GCs exert their effects through binding to the glucocorticoid receptor (GR), a member of the nuclear receptor superfamily of transcription factors. In the hepatocytes, GCs have been shown to enhance gluconeogenesis and hepatic glucose production through direct recruitment to the promoter region of gluconeogenic genes and indirect mechanisms involving the induction of Krüppel-like factor 9 [[13], [14], [15], [16], [17]]. However, in contrast to the well-defined molecular pathways regulating glucose metabolism, the molecular basis of GCs in the development of fatty liver remains poorly understood.

In the present study, we show that treatment of dexamethasone (DEX), a synthetic analog of GCs, resulted in TGs accumulation in the livers of healthy mice, but not in the cultured hepatocytes. We further show that DEX could upregulate the expression levels of Periostin in white adipose tissues, which in turn contributes to liver steatosis and hyperglycemia.

## 2 Materials and methods

### 2.1 Mouse experiments

C57BL/6 male mice aged 8–10 weeks were purchased from Shanghai Laboratory Animal Company (SLAC, Shanghai, China). Periostin knockout mice were obtained from Dr. Yan Lu (Zhongshan Hospital, Fudan University, China) [18]. To block the role of Periostin, the Periostin antibody was injected at a dose of 5 mg/kg into mice [18]. All mice were housed in a temperature- and light-controlled environment with a 12-h light and 12-h dark cycle. For DEX treatment, mice received daily intraperitoneal injections of dexamethasone (DEX, Sigma–Aldrich, 2.5 mg/kg) or saline for 14 days. Blood glucose was measured using a portable blood glucose meter (LifeScan, Johnson & Johnson, New Jersey, USA). Plasma levels of insulin and corticosterone were determined using commercial kits from Millipore (Merck, USA). For insulin tolerance tests (ITTs), mice were injected with regular human insulin (Eli Lily, Indianapolis, Indiana, USA) at a dose of 0.75 U/kg body weight after a 6-h fast. The animal protocol was reviewed and approved by the animal care committees of Shanghai Pudong New Area People's Hospital and XinHua Hospital.

### 2.2 Cell culture

Human embryonic kidney cells (HEK293T cells) and hepatocellular carcinoma cells (Hep1-6 and HepG2 cells) were purchased from Cell Bank of Shanghai Institute of Biological Science (SIBS, CAS, Shanghai, China). Cells were cultured in Dulbecco's modified Eagle's medium (DMEM) (Invitrogen), supplemented with 10% FBS (Gibco), 100 units/mL penicillin, and 100 mg/mL streptomycin (Gibco). 3T3-L1 fibroblasts were obtained from American Type Culture Collection (Rockville, MD) and were cultured and differentiated into adipocytes as described previously with some modifications [19]. Briefly, the fibroblasts were grown in DMEM supplemented with 100 U/ml penicillin, 100 μg/ml streptomycin, and 10% fetal bovine serum (FBS). After confluence, the fibroblasts were maintained for another 2 days (from day 0). Differentiation was induced by treating the cells with standard differentiation inducers (DMEM containing 0.5 mM IBMX, 1 lM dexamethasone, and 10 lg/ml insulin and 10% FBS) for 48 h (from day 0 to day 2). The cells were re-fed with DMEM supplemented with 10 lg/ml insulin and 10% FBS for the following 48 h (from day 2 to day 4), then the medium was replaced by growth medium, changed every 2 days. The differentiated cells were used in experiments until more than 90% of cells were demonstrating adipocyte morphology. C2C12 cells were cultured in DMEM supplemented with 5 mM glucose, 20% FBS, 2 mM glutamine, and penicillin/streptomycin. Upon reaching confluence, cells were cultured in DMEM containing 25 mM glucose, 2% horse serum, 2 mM glutamine, and penicillin/streptomycin.

### 2.3 Hepatic and cellular TG measurement

Cultured cells were harvested using a cell scraper and homogenized by sonication. For determination of lipid contents, extracts were obtained from cell homogenates or liver tissues using a methanol–chloroform mixture by the Folch method [20]. After evaporation of the organic solvent, the triglyceride (TG) content of each sample was measured using the TG measurement reagent from BioVision Company (Milpitas, CA, USA) according to the manufacturer's instructions.

### 2.4 RNA isolation and quantitative real-time PCR

Total RNAs were isolated from tissues or cells using TRIzol (Invitrogen) according to the manufacturer's instructions. For quantification of the transcripts of the interest genes, real-time PCR was performed using SYBR Green Premix Ex Taq (Takara Bio, Otsu, Japan) on Light Cycler 480 (Roche, Basel, Switzerland). The sequences of all used primers are available upon request. For the quantitative real-time PCR analysis, we determined the threshold cycles (CT) values using fixed threshold settings after the initial cycling was completed. The PCR amplification efficiencies of these primers were between 90% and 110%.

### 2.5 Western blotting and antibodies

Homogenized tissues and cells were lysed in RIPA buffer containing 1 × PBS, 1% NP40, 5 mM EDTA, 0.1% sodium dodecyl sulfate (SDS), 1 mM Na_3_VO_4_, 1% phenylmethanesulfonylfluoride, complete protease inhibitor cocktail (Sigma) and complete phosphatase inhibitors. The lysates were centrifuged at 12,000 g for 10 min at 4 °C to remove the insoluble materials, and the supernatants were boiled in SDS loading buffer. The boiled samples were separated by 10% SDS–polyacrylamide gel and electroblotted to nitrocellulose transfer membranes (Whatman, GE Healthcare). The membranes were blocked with 5% milk and incubated with different antibodies, followed by incubation with secondary antibodies. The primary antibodies used in western blotting included anti-PPARα (Millipore), anti-Periostin (Sigma-aldrich) and anti-β-actin (Abcam). Periostin neutralizing antibody was obtained from Dr. Yan Lu (Zhongshan Hospital, Fudan University, China) [18]

### 2.6 Transfection and dual-luciferase reporter assay

The promoter regions of mouse *Postn* gene were amplified from the mouse genomic DNA template and inserted into pGL4.15 empty vectors (Promega). Mutant promoters were generated using a PCR mutagenesis kit (Toyobo). All of the transient transfections were conducted using Lipofectamine 2000 (Invitrogen) according to the manufacturer's instructions. For luciferase reporter assays, cells were seeded in 24-well plates and transfected with the indicated plasmids. Renilla luciferase pRL-SV40 (Promega) was used to normalize the luciferase activity, which was further measured using the dual-luciferase reporter assay system (Promega).

### 2.7 Chromatin immunoprecipitation

A chromatin immunoprecipitation (ChIP) assay kit was used (Upstate Biotechnology). In brief, lysates from 3T3-L1 cells were fixed with formaldehyde. DNA was sheared to fragments at 200–1,000 bp using sonication. The chromatins were incubated and precipitated with antibodies against GR (Santa Cruz Biotechnology), or IgG (Santa Cruz Biotechnology).

### 2.8 Statistical analysis

All values are presented as means SEM. Statistical differences were determined by a Student *t* test. Statistical significance is displayed as * *P* < 0.05, **P < 0.01, or ***P < 0.001.

## 3 Results

### 3.1 Dexamethasone treatment induces liver steatosis in mice but not in cultured hepatocytes

**Figure 1:**
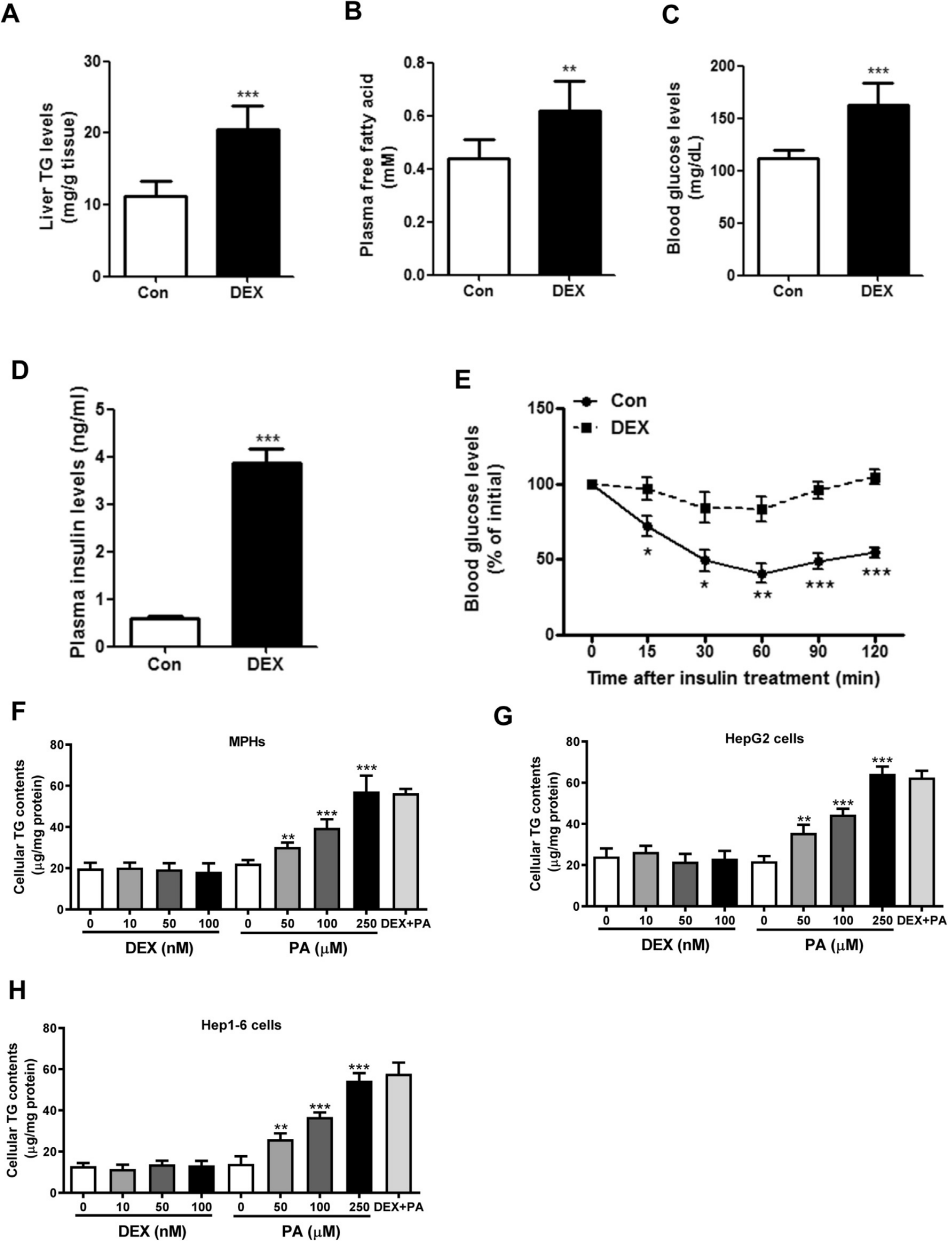
**DEX induces liver steatosis in mice but not in the hepatocytes.** (A–E): C57BL/6 male mice were treated with dexamethasone (DEX, 2.5 mg/kg) or vehicle control (Con) for 14 days. Hepatic triglycerides (TG) contents (A), plasma free fatty acids levels (B), blood glucose levels (C), plasma insulin levels (D) and insulin tolerance tests (E) were determined. n = 8 per group. (F–H) Cellular TG contents were measured in mouse primary hepatocytes (MPHs) (F), HepG2 cells (G) and Hep1-6 cells (H) treated with DEX (0, 10, 50, 100 nM) or palmitate (PA, 0, 50, 100, 250 μM) or DEX plus PA (100 nM plus 250 μM). n = 4 per group. *P < 0.05,**P < 0.01, ***P < 0.001.

To determine the role of GCs in liver steatosis, we firstly injected C57BL/6 male mice with Dexamethasone (DEX) or saline as a vehicle control for 14 days. As expected, DEX treatment dramatically increased hepatic triglycerides (TGs) and plasma free fatty acids (FFAs) in mice (Figure 1A–B). Besides, blood glucose and insulin levels were increased while insulin sensitivity were reduced as shown by insulin tolerance tests (Figure 1C–E).

To further test the role of DEX in hepatic steatosis *in vitro*, mouse primary hepatocytes (MPHs), HepG2 cells and Hep1-6 cells were cultured with different dosage of DEX for 24 h. Palmitate acid (PA), a saturated fatty acid commonly found in both obese animals and humans (17), were used as a positive control. As a result, PA, but not DEX treatment, led to an increased TG accumulation in three types of hepatocytes (Figure 1F–H). Besides, combination of PA and DEX did not enhance the steatotic role of PA. Therefore, our results suggest that the steatotic roles of DEX might be distinct *in vivo* and *in vitro*.

### 3.2 DEX inhibits fatty acid β-oxidation in mice but not in hepatocytes

**Figure 2:**
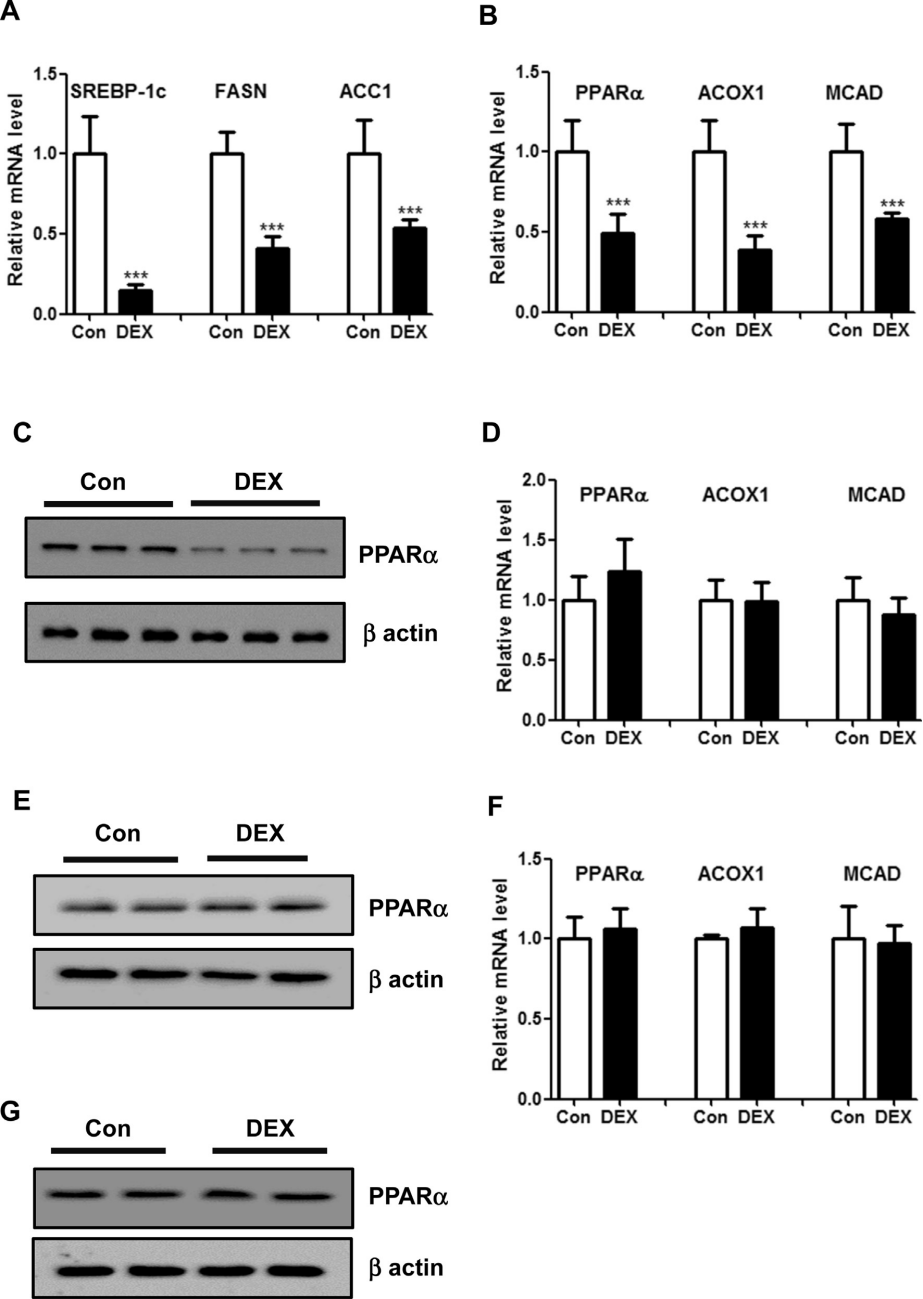
**DEX inhibits fatty acid β-oxidation in the livers of mice but not in hepatocytes.** (A–B) Relative mRNA levels of genes related to de novo lipogenesis (A) and fatty acid β-oxidation (B) in the livers of mice treated with DEX or vehicle control. n = 8 per group. (C) Representative protein levels of PPARα in the livers of mice treated with DEX or vehicle control. (D) Relative mRNA levels of genes related to fatty acid β-oxidation in MPHs treated with DEX (100 nM) or vehicle control. n = 5 per group. (E) Representative protein levels of PPARα MPHs treated with DEX or vehicle control. (F) Relative mRNA levels of genes related to fatty acid β-oxidation in HepG2 cells treated with DEX (100 nM) or vehicle control. n = 5 per group. (G) Representative protein levels of PPARα in HepG2 cells treated with DEX or vehicle control. ***P < 0.001.

To explore the molecular mechanism of DEX-induced liver steatosis, genes expression analysis were performed by quantitative real-time PCR. We first examined TAT, the glucocorticoid receptor (GR) target gene, and found that DEX significantly upregulated the expression of TAT in the livers of C57BL/6 mice (Supplementary Fig. 1A). PEPCK and G6Pase, two key enzymes involved in gluconeogenesis, were also upregulated in response to DEX treatment (Supplementary Fig. 1B). Furthermore, we analyzed the expression levels of genes involved in de novo lipogenesis and fatty acid β-oxidation. As a result, mRNA levels of lipogenic genes, including SREBP-1c, FASN and ACC1, were reduced (Figure 2A), suggesting that lipogenesis cannot account for the fatty liver induced by DEX. On the other hand, fatty acid β-oxidation-related genes, including PPARα and its down-stream target genes (ACOX1 and MCAD), were significantly inhibited upon DEX treatment (Figure 2B). The downregulation of hepatic PPARα by DEX treatment were also confirmed by western blots (Figure 2C). Therefore, our results suggest that suppression of fatty acid β-oxidation contributes to the hepatic steatosis induced by DEX.

We further examined the expression levels of genes above mentioned in cultured hepatocytes. In agreement, DEX treatment dramatically upregulated the expression of TAT, PEPCK and G6Pase in MPHs and HepG2 cells (Supplementary Figs. 1C–1F). However, PPARα and its down-stream target genes were not altered (Figure 2D–G, Supplementary Figs. 1G–1H). Taken together, our data indicate that systemic factors derived from other tissues may mediate the steatotic roles of DEX.

### 3.3 Adipokines mediates the hepatic steatotic roles of DEX

**Figure 3:**
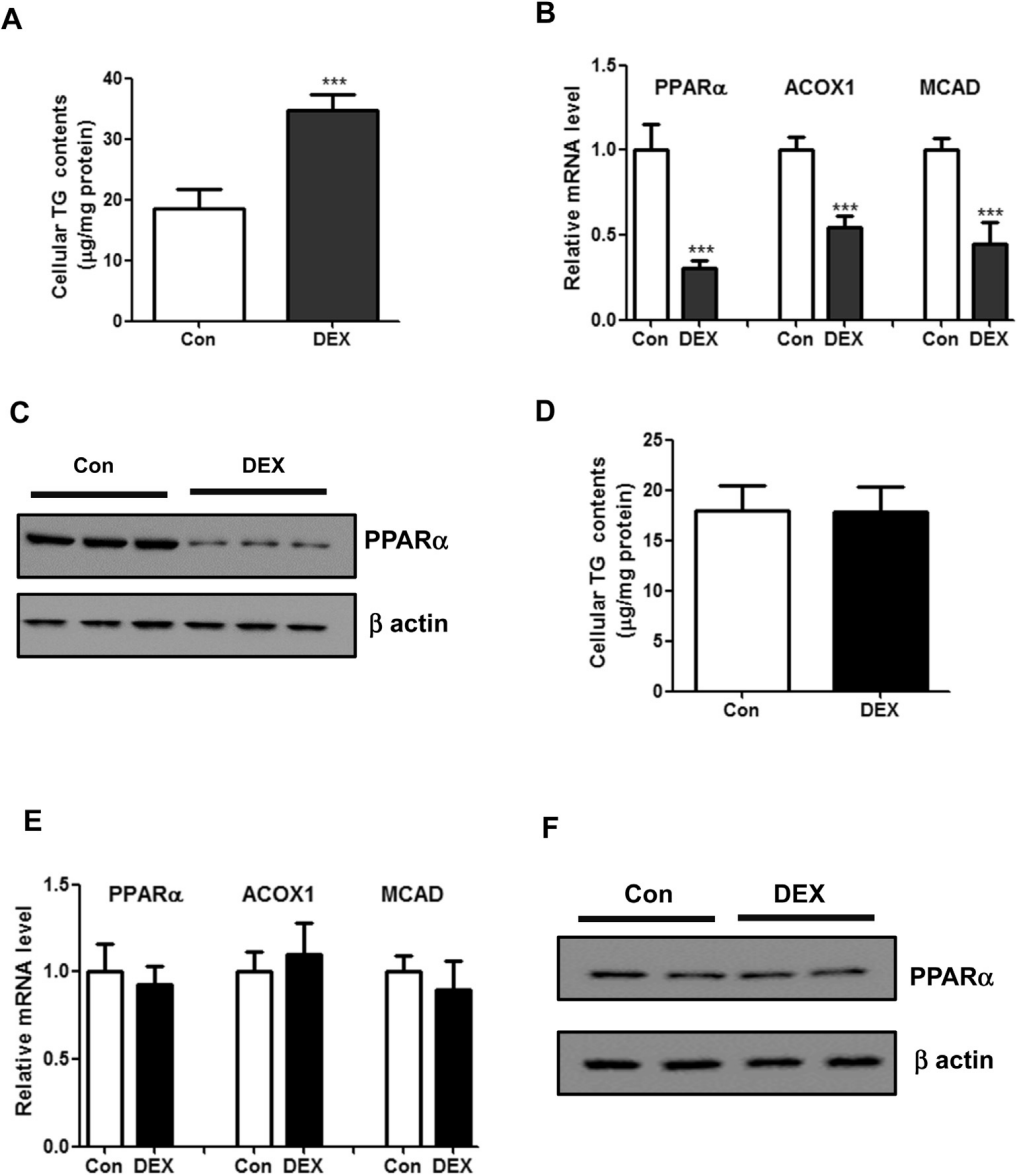
**Adipokines potentially mediate the hepatic steatotic roles of DEX.** (A–C): 3T3-L1 preadipocytes were differentiated into mature adipocytes and then treated with DEX for 48 h. Then, the supernatants were collected to treat the mouse primary hepatocytes (MPHs) for 48 h. Cellular TG contents (A), mRNA levels of genes related to fatty acid β-oxidation (B) and protein levels of PPARα (C) were determined. n = 4 per group. (D–F): C2C12 cells were differentiated into mature skeletal muscle cells and then treated with DEX for 48 h. Then, the supernatants were collected to treat the mouse primary hepatocytes (MPHs) for 48 h. Cellular TG contents (A), mRNA levels of genes related to fatty acid β-oxidation (B) and protein levels of PPARα (C) were determined. n = 4 per group. ***P < 0.001.

Recent studies have shown that adipokines derived from adipose tissues and myokines derived from skeletal muscles play important roles in the development of fatty liver [8,10,21]. Therefore, to clarify whether adipokines and/or myokines mediate the hepatic steatotic roles of DEX, 3T3-L1 cells and C2C12 cells were successfully differentiated into adipocytes and skeletal muscle cells, respectively. Then, both cells were treated with DEX for 48 h and the supernatants were collected to treat the mouse primary hepatocytes (MPHs). As a result, we found that the supernatants collected from 3T3-L1 mature adipocytes could induce TG accumulation and downregulate fatty acid oxidation-related genes in MPHs (Figure 3A–C). On the other hand, the supernatants collected from C2C12 cells could not alter TG contents and PPARα expression in MPHs (Figure 3D–F), suggesting that adipokines induced by DEX could mediate the hepatic steatotic roles of DEX.

### 3.4 DEX upregulates Periostin expression in adipocytes

**Figure 4:**
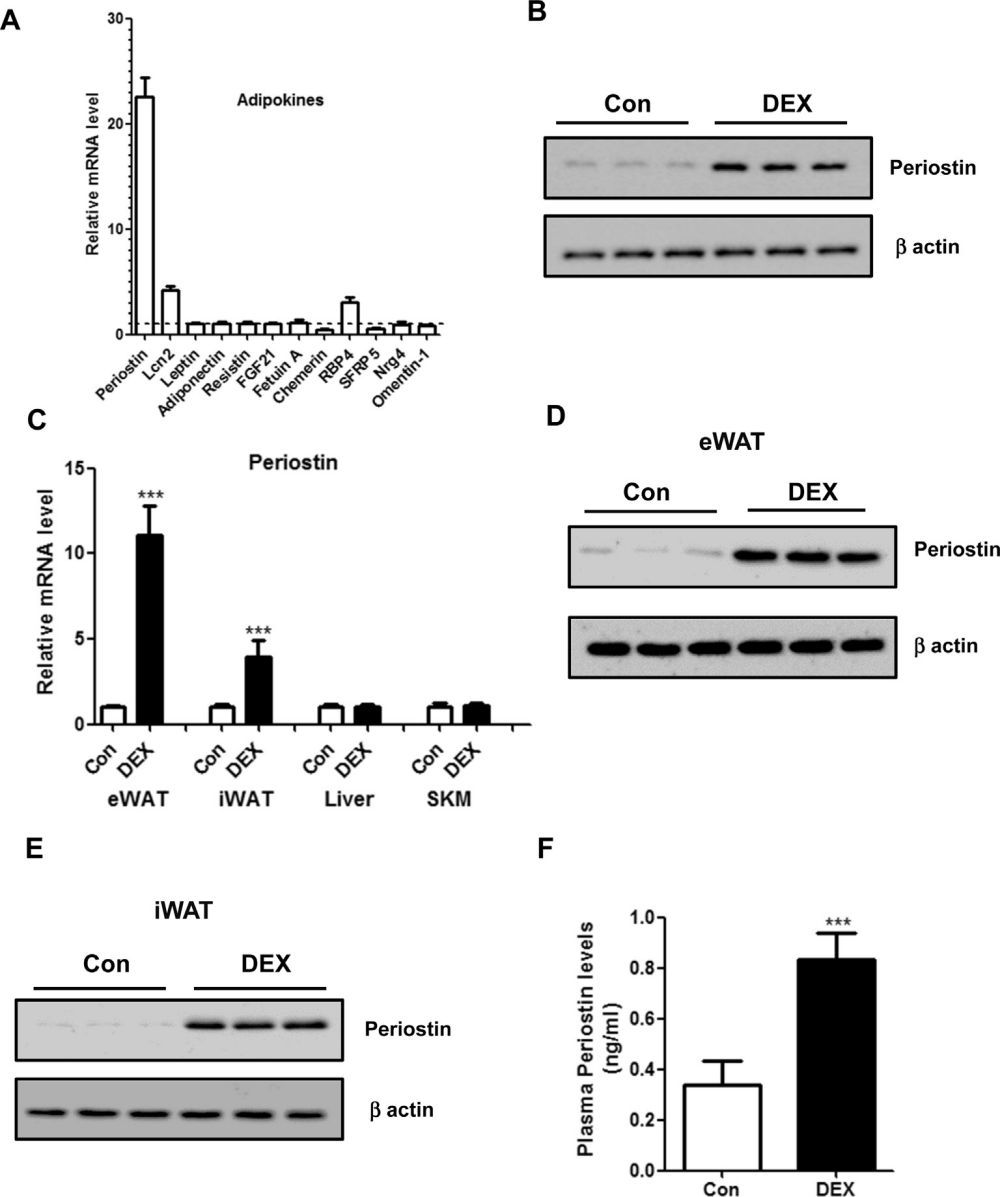
**DEX upregulates Periostin expression in adipocytes.** (A) 3T3-L1 mature adipocytes were treated with DEX (100 nM) or vehicle control for 24 h. Then, the transcript levels of several adipokines were measured by quantitative real-time PCR, normalized to vehicle control and depicted as a fold change between DEX and controls (set as 1, dotted line). n = 5 per group. (B) Protein levels of Periostin were determined in 3T3-L1 adipocytes treated with DEX or vehicle control. (C) Relative mRNA levels of Periostin in several metabolic tissues from mice treated with DEX or vehicle control. eWAT: epididymal white adipose tissue; iWAT: inguinal white adipose tissue; SKM: skeletal muscle. n = 8 per group. (D–E) Protein levels of Periostin were analyzed in eWAT (D) and iWAT (E) from two groups of mice. (F) Plasma Periostin levels were measured by ELISA assays from two groups of mice. ***P < 0.001.

To further uncover which adipokine mediates the hepatic steatotic roles of DEX, quantitative real-time PCR analysis was performed to detect the mRNA expression of adipokines. Notably, we found that Periostin was dramatically induced by DEX in 3T3-L1 mature adipocytes (Figure 4A), which was further confirmed by western blots (Figure 4B). Recent studies have shown that Periostin could promote liver steatosis and through suppression of PPARα and fatty acid β-oxidation (18). Therefore, Periostin was chosen for further studies. We found a dose-dependent induction of Periostin mRNA expression in 3T3-L1 adipocytes (Supplementary Fig. 2A). Using the moderate dose of DEX (100 nM), we examined induction at different time points ranging from 2 to 36 h. At 2 h after treatment, there was no increase in Periostin mRNA expression following DEX treatment. However, at 4 h, expression is significantly increased over baseline levels with a highest rise in expression found at the 24 h time point (Supplementary Fig. 2B). The induction of Periostin by DEX was largely blocked by RU486, a GR antagonist, suggesting that the action of DEX on the expression of Periostin is likely direct via GR (Supplementary Fig. 2C).

To test this regulatory role *in vivo*, the mRNA expression Periostin from epididymal white adipose tissues (eWAT), inguinal white adipose tissues (iWAT), livers and skeletal muscles were examined. As a result, its expression in eWAT and iWAT, but not in the livers and skeletal muscles, were significantly increased by DEX (Figure 4C–E). Consistently, a significant increase of plasma Periostin concentrations was observed in DEX-treated mice (Figure 4F).

To further confirm the role of GCs in the regulation of Periostin expression, we examined the rhythm of plasma GC levels and Periostin expression. As expected, we found a similar pattern form plasma corticosterone levels and Periostin mRNA levels in C57BL/6 mice (Supplementary Figs. 3A–3B). In addition, patients with Cushing syndrome, which is characterized by elevated endogenous GCs, were recruited. Indeed, plasma GC levels were dramatically increased in Cushing patients and plasma Periostin levels were also increased, compared with normal subjects (Supplementary Figs. 4A–4B). Moreover, plasma GC levels were positively correlated with plasma Periostin levels in this human cohort (Supplementary Fig. 4C). Taken together, these data demonstrated that Periostin could represent an important downstream adipokine for GCs.

### 3.5 Periostin mediates the roles of DEX to induce liver steatosis

**Figure 5:**
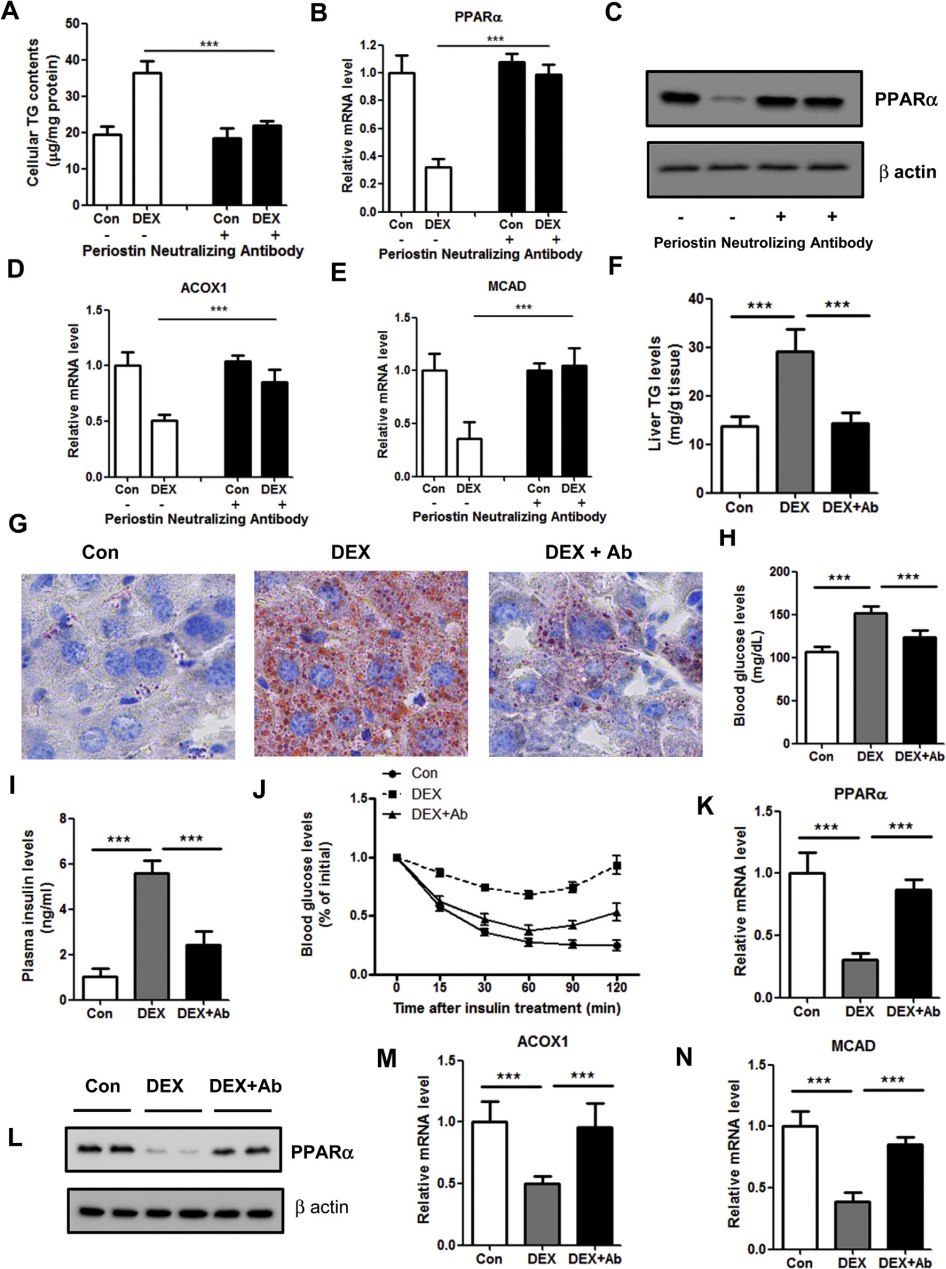
**Neutralizing of Periostin attenuated the hepatosteatosis induced by DEX.** (A–E): 3T3-L1 preadipocytes were differentiated into mature adipocytes and then treated with DEX for 48 h. Mouse primary hepatocytes (MPHs) were pre-incubated with Periostin neutralizing antibody for 2 h and then treated with the supernatants from 3T3-L1 cells for 48 h. Cellular TG contents (A), mRNA and protein levels of PPARα (B, C), expression of genes related to fatty acid β-oxidation (D, E) were determined. n = 4 per group. (F–N) C57BL/6 male mice were treated with DEX (2.5 mg/kg), DEX (2.5 mg/kg) plus Periostin antibody (5 mg/kg) or vehicle control. Liver TG contents (F, G), blood glucose levels (H), plasma insulin levels (I), insulin tolerance test (J), mRNA and protein levels of PPARα (K, L), expression levels of ACOX1 and MCAD (M, N) were analyzed in three groups of mice. n = 6 per group. ***P < 0.001.

To determine the contribution of Periostin to the steatotic roles of GCs, two approaches were used. Firstly, MPHs were pre-incubated with Periostin neutralizing antibody and then administrated with the supernatants from DEX-treated 3T3-L1 mature adipocytes. As a result, Periostin antibody significantly attenuated the TG accumulation induced by the supernatants of 3T3-L1 cells (Figure 5A). Consistently, down-regulation of PPARα and its target genes, was also blocked by Periostin antibody (Figure 5B–E). Similar results were also obtained in HepG2 cells (Supplementary Figs. 5A–5E). In addition, administration of Periostin antibody into DEX-treated 3T3-L1 cells also attenuated the TG accumulation and downregulation of PPARα in MPHs (Supplementary Figs. 5F and 5G).

Secondly, Periostin neutralizing antibody significantly reduced the DEX-induced hepatic TG retention in C57BL/6 mice (Figure 5F–G). Besides, DEX-induced hyperglycemia, hyperinsulinemia and insulin resistance were also partially reversed by Periostin antibody (Figure 5H–J). Consistently, downregulation of PPARα and its target genes was blocked by Periostin antibody (Figure 5K-5N). Therefore, these results demonstrated that blocking the roles of Periostin with a neutralizing antibody can improve liver steatosis in DEX-treated mice, suggesting that Periostin mediates the roles of DEX to induce hepatosteatosis.

### 3.6 Periostin knockout mice are resistant to DEX-induced hepatosteatosis

**Figure 6:**
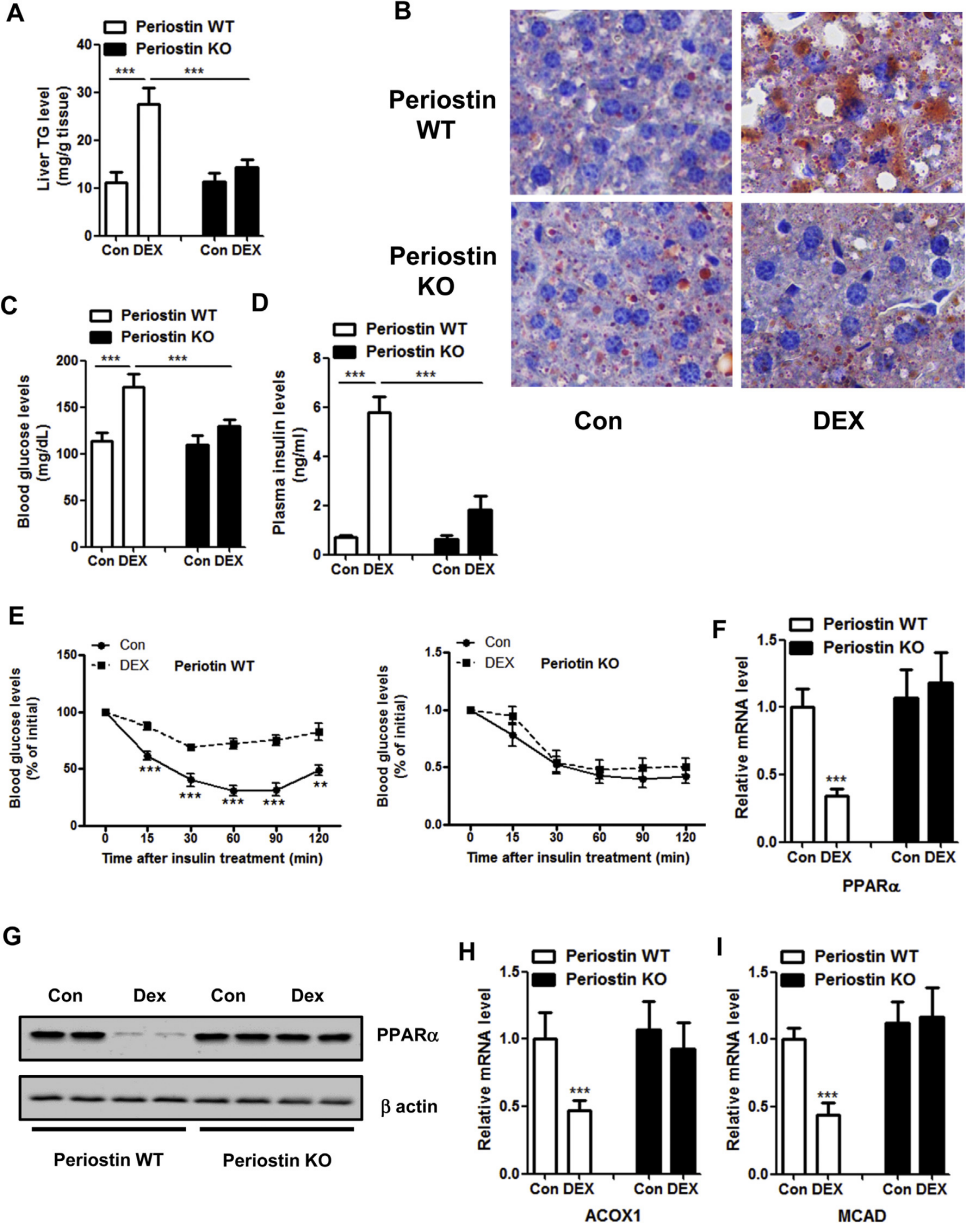
**Periostin knockout mice are protected from DEX-induced hepatic steatosis.** (A–I) Periostin wild-type and knockout mice were treated with DEX (2.5 mg/kg) or vehicle control for 14 days. Liver TG contents (A, B), blood glucose levels (C), plasma insulin levels (D), insulin tolerance tests (E), mRNA and protein levels of PPARα (F, G), expression levels of ACOX1 and MCAD (H, I) were analyzed. n = 8 per group. **P < 0.01, ***P < 0.001.

To explore whether Periostin knockout mice are resistant to the metabolic side effects of GCs, wild-type and Periostin knockout mice were treated with DEX or vehicle control. Periostin knockouts have been shown reduced body weight due to postnatal growth retardation and skeletal defects, which is also observed in our study (Supplementary Fig. 6A). DEX did not affect body weight and food intake in two groups of mice (Supplementary Figs. 6A–6B). As a results, the DEX-treated wild-type mice developed hepatosteatosis, hyperglycemia, hyperinsulinemia and insulin resistance (Figure 6A–E). However, DEX-treated Periostin knockout mice were refractory to these effects (Figure 6A–E). In addition, DEX could suppress the expression of PPARα and its target genes in wild-type mice, but not in the Periostin knockout mice (Figure 6F–I). Therefore, our results indicate that Periostin is required for GCs-induced hepatosteatosis and hyperglycemia in mice.

### 3.7 DEX upregulates Periostin expression through two functional GR-response elements

**Figure 7:**
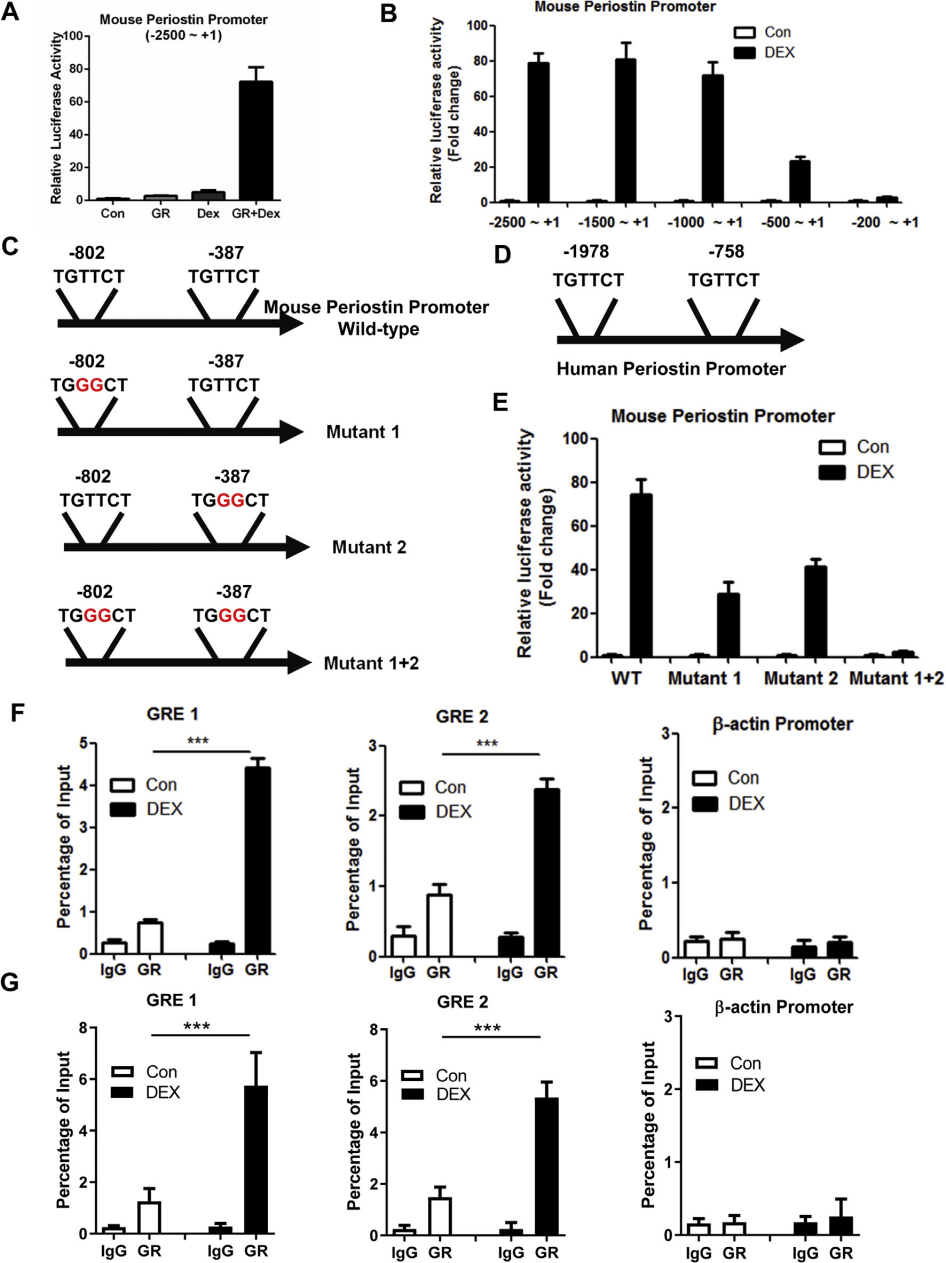
**DEX upregulates the Periostin promoter through two half GREs.** (A) The transcriptional activity of the full-length mouse Periostin promoter (−2500 to +1) was upregulated by GR in the presence of DEX (100 nM). HEK293T cells were co-transfected with GR expression plasmid. (B) The transcriptional activity of truncated Periostin promoters in HEK293T cells overexpressing GR. (C) The mouse Periostin promoter contains two potential half GR response elements (GREs). Point mutations were highlighted in red. (D) The human Periostin promoter contains two potential half GR response elements (GREs). (E) The transcriptional activity of wild-type or mutant Periostin promoters in HEK293T cells overexpressing GR. (F–G) Chromatin immunoprecipitation (ChIP) assays showing the recruitment of GR onto the promoter regions of Periostin in 3T3-L1 adipocytes (F) or eWAT (G) of C57BL/6 mice treated with DEX. The proximal promoter region of β actin gene was used as a negative control for ChIP assays.

Finally, to elucidate whether GR directly regulates Periostin expression, we analyzed the transcriptional activity of mouse Periostin promoter. We generated a luciferase reporter containing the promoter region from position −2500 bp to +1 bp (translational start site). The luciferase reporter assays showed that the transcriptional activity of the Periostin promoter was dramatically increased by GR in the presence of DEX (Figure 7A). Subsequently, through a series of truncated promoters, we defined two GR-responsive regions from −1000 bp to −500 bp and −500 bp to −200 bp (Figure 7B). We further found two half-GR response elements (GREs) in these regions (Figure 7C). Two potential half-GREs were also found in human Periostin promoter (Figure 7D). Mutation of either GRE reduced, while mutation of both GREs could completely blocked the activity of Periostin promoter by DEX (Figure 7E). Moreover, chromatin immunoprecipitation (ChIP) assays revealed that upon DEX treatment, GR could bind to these two GREs in 3T3-L1 mature adipocytes and eWAT of C57BL/6 mice (Figure 7F–G). Together, our results suggest that DEX upregulates Perostin expression through two functional GREs in adipocytes.

## 4 Discussion

Our present findings showed that chronic DEX treatment dramatically increased hepatic TG contents *in vivo*, which was attributed to suppression of fatty acid β-oxidation. However, using three types of hepatocytes, we unexpectedly found that DEX treatment did not led to an increased TG accumulation and inhibition of fatty acid β-oxidation *in vitro*. Therefore, our data suggest that systemic factors derived from other tissues may mediate the steatotic roles of DEX. We further found that DEX could upregulate the expression levels of Periostin in white adipose tissue, which in turn induced liver steatosis. In agreement, both administration of a Periostin-neutralizing antibody and genetic ablation of Periostin largely attenuated DEX-induced hepatic steatosis, hyperglycemia and insulin resistance, indicating that Periostin plays an important role in the GCs-induced metabolic side effects.

Although it has been well-established that long-term GCs therapy is associated with hepatic steatosis [11,12], the molecular mechanisms remain controversial. Lemke U et al. showed that liver-specific knockdown of GR improved hepatosteatosis in leptin receptor deficient obese mice through regulation of transcriptional repressor hairy enhancer of split 1 (Hes1) gene expression [22]. However, this result was not observed in another study [23]. Besides, alleviation of liver steatosis by depletion of hepatic GR might be secondary to the improvement by hyperglycemia and insulin resistance. Indeed, a recent study generated a mouse model of conditional GR invalidation specifically in adipose tissues of adult mice (AdipoGR-KO mice) and these mice were protected from lipid ectopic deposition in liver [24], highlighting the importance roles of adipocyte GR in the regulation of hepatic steatosis.

Periostin (encoded by *Postn*) is a secreted cell adhesion protein belonging to the fasciclin family [25]. Previous studies have shown that Periostin is involved in the development of multiple tumors via several signaling pathways, such as PI3K/AKT and Wnt/β-catenin [[26], [27], [28], [29], [30]]. Periostin expression is markedly upregulated in various human tumors, including in head and neck, breast, colon, pancreatic, and ovarian cancers [28,31]. Recent studies showed that Periostin is also involved in metabolic dysfunction. Lu et al. showed that Periostin plays an important role in liver steatosis and hypertriglyceridemia [18]. Consistently, we and others found that elevated circulating Periostin level was associated with an increased risk of developing nonalcoholic fatty liver disease [18,[32], [33], [34]]. In adipose tissue, loss of Periostin occurs in aging adipose tissue of mice and its genetic ablation impairs adipose tissue lipid metabolism [35]. Together with these studies, our findings support the notion that Periostin derived from other tissues, such as adipocytes, could contribute to circulating Periostin and promote metabolic disorders.

There are still several issues to be clarified. For instance, GCs could decrease the expression of inflammatory cytokines such as interleukin-6 (IL-6) and tumor necrosis factor alpha (TNFα) while Periostin was shown to plays a critical role in the amplification and chronicity of inflammation [[36], [37], [38], [39], [40]]. Besides, chronic GC treatment is associated with osteoporosis, while Periostin could promote bone regeneration [41]. Therefore, it is tempting to assume that partial effects of GCs might be dissociated with Periostin. Besides, given that Periostin is ubiquitously expressed, whether GCs could regulate Periostin expression in other tissues remains to be determined. Adipocyte-specific Periostin knockout mice will further help to clarify this issue.

Together, our findings provided a novel insight that GCs could promote liver steatosis through integrative organ crosstalk mediated by systemic factors. Periostin might therefore be a therapeutic target for GCs induced fatty liver and related metabolic side effects.

## Author's contributions

J.W. and Y.S. gathered the data and wrote the manuscript, S.X., S.C. and X.L. provided technical assistance for the study, J.J.,Q.S. and B.L., provided critical comments on the manuscript, W.S. and B.L., directed the project, reviewed and edited the manuscript.

## Appendix A Supplementary data

Multimedia component 1 **S1:** (A-B) Relative mRNA levels of TAT and gluconeogenic genes (PEPCK, G6Pase) in the livers of mice treated with DEX or vehicle control. (C-D) Relative mRNA levels of TAT and gluconeogenic genes in MPHs treated with DEX or vehicle control. (E-F) Relative mRNA levels of TAT and gluconeogenic genes in HepG2 treated with DEX or vehicle control. (G-H) Relative mRNA levels of PPARα in MPHs and HepG2 cells treated with DEX or vehicle control for different hours. **S2:** (A) Relative mRNA levels of Periostin in 3T3-L1 mature adipocytes treated with different dose of DEX. (B) Relative mRNA levels of Periostin in 3T3-L1 mature adipocytes treated with DEX or vehicle control for different hours. (C) Relative mRNA levels of Periostin in 3T3-L1 mature adipocytes treated with DEX, in the absence or presence of RU486. **S3:** Circadian alteration of plasma corticosterone levels (A) and Periostin mRNA expression (B) in adipose tissues at the indicated time points in C57/BL6 mice. n=7 per group. **S4:** Plasma cortisol (A) and Periostin concentrations (B) in normal subjects and patients with Cushing syndrome. (C) Pearson correlation of plasma cortisol and Periostin levels in two groups of subjects. **S5:** (A-E): 3T3-L1 preadipocytes were differentiated into mature adipocytes and then treated with DEX for 48 h. HepG2 were pre-incubated with Periostin neutralizing antibody for 2 hr and then treated with the supernatants from 3T3-L1 cells. Cellular TG contents (A), mRNA and protein levels of PPARα (B, C), expression of genes related to fatty acid β-oxidation (D, E) were determined. (F-G) 3T3-L1 preadipocytes were differentiated into mature adipocytes. Then, cell were treated with DEX and Periostin antibody for 48 hr and 2 hr, respectively. Mouse primary hepatocytes (MPHs) were treated with the supernatants from 3T3-L1 cells for 48 hr. Cellular TG contents (F) and mRNA levels of PPARα (G) were determined. n=4 per group. **S6:** (A-B) Body weight and food intake of wild-type and Periostin knockout mice treated with DEX or vehicle control. n=8 per group. Multimedia component 1
