# Supplementary material for: Adipocyte-derived Periostin mediates glucocorticoid-induced hepatosteatosis in mice
Source: Mol Metab. 2019 Nov 9;31:24–35. doi: 10.1016/j.molmet.2019.11.003 (PMC6880106; doi:10.1016/j.molmet.2019.11.003)
Supplement: Multimedia component 1 — S1: (A-B) Relative mRNA levels of TAT and gluconeogenic genes (PEPCK, G6Pase) in the livers of mice treated with DEX or vehicle control. (C-D) Relative mRNA levels of TAT and gluconeogenic genes in MPHs treated with DEX or vehicle control. (E-F) Relative mRNA levels of TAT and gluconeogenic genes in HepG2 treated with DEX or vehicle control. (G-H) Relative mRNA levels of PPARα in MPHs and HepG2 cells treated with DEX or vehicle control for different hours. S2: (A) Relative mRNA levels of Periostin in 3T3-L1 mature adipocytes treated with different dose of DEX. (B) Relative mRNA levels of Periostin in 3T3-L1 mature adipocytes treated with DEX or vehicle control for different hours. (C) Relative mRNA levels of Periostin in 3T3-L1 mature adipocytes treated with DEX, in the absence or presence of RU486. S3: Circadian alteration of plasma corticosterone levels (A) and Periostin mRNA expression (B) in adipose tissues at the indicated time points in C57/BL6 mice. n=7 per group. S4: Plasma cortisol (A) and Periostin concentrations (B) in normal subjects and patients with Cushing syndrome. (C) Pearson correlation of plasma cortisol and Periostin levels in two groups of subjects. S5: (A-E): 3T3-L1 preadipocytes were differentiated into mature adipocytes and then treated with DEX for 48 h. HepG2 were pre-incubated with Periostin neutralizing antibody for 2 hr and then treated with the supernatants from 3T3-L1 cells. Cellular TG contents (A), mRNA and protein levels of PPARα (B, C), expression of genes related to fatty acid β-oxidation (D, E) were determined. (F-G) 3T3-L1 preadipocytes were differentiated into mature adipocytes. Then, cell were treated with DEX and Periostin antibody for 48 hr and 2 hr, respectively. Mouse primary hepatocytes (MPHs) were treated with the supernatants from 3T3-L1 cells for 48 hr. Cellular TG contents (F) and mRNA levels of PPARα (G) were determined. n=4 per group. S6: (A-B) Body weight and food intake of wild-type and Periostin knockout mice tre [file mmc1.pptx]

## Slide 1
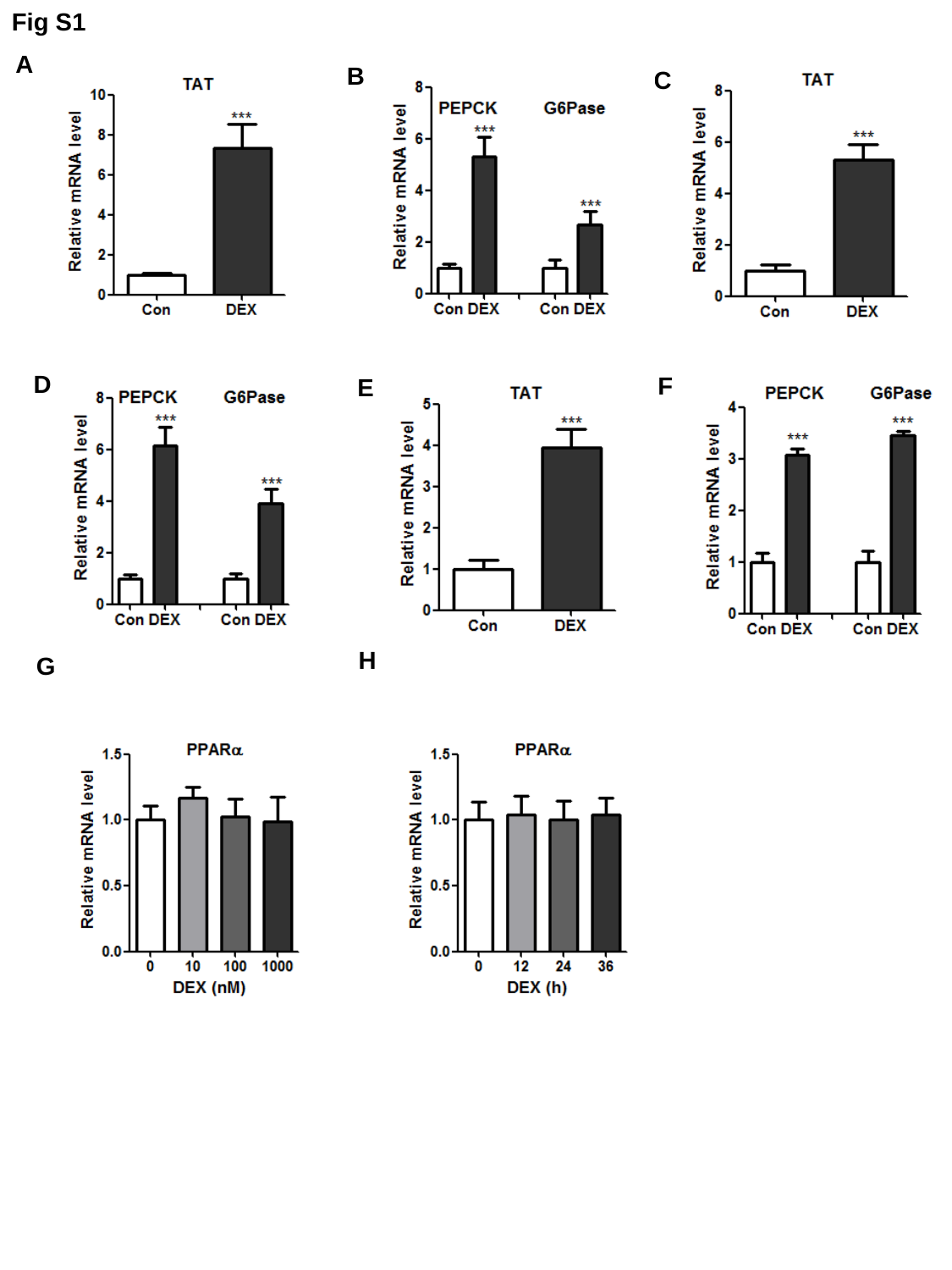

Fig S1
A
B
C
D
F
E
H
G

## Slide 2
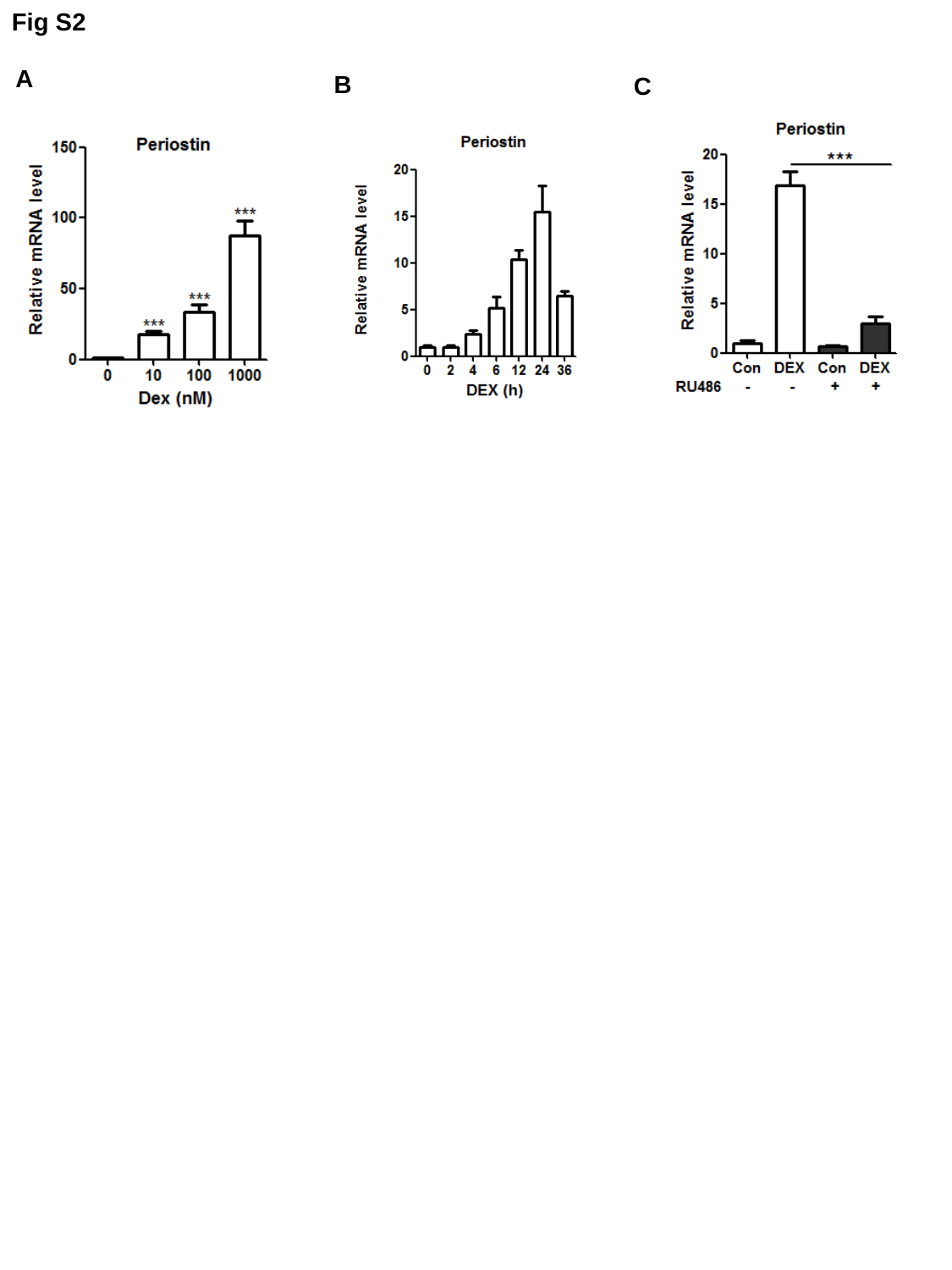

Fig S2
A
B
C

## Slide 3
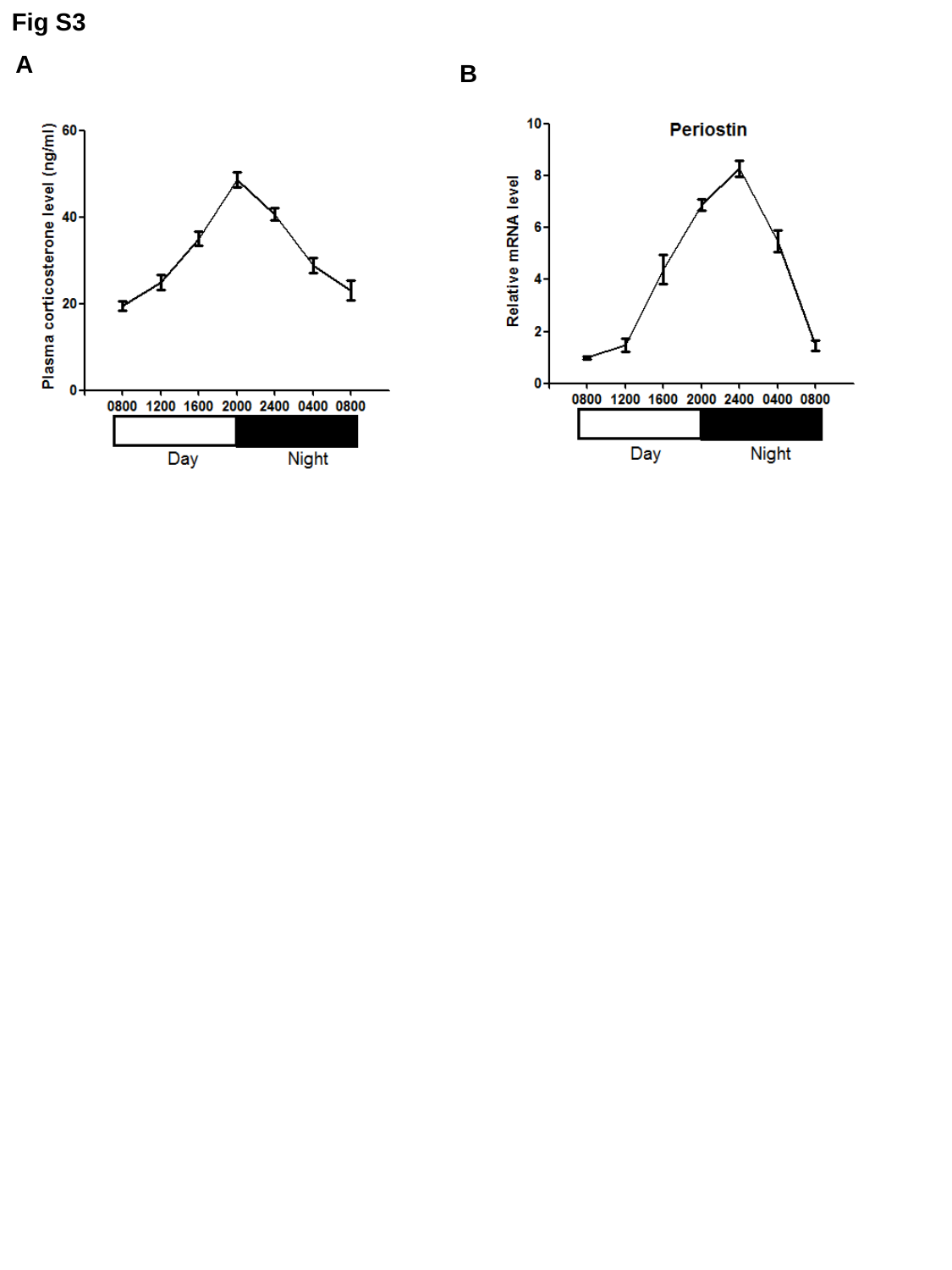

Fig S3
A
B

## Slide 4
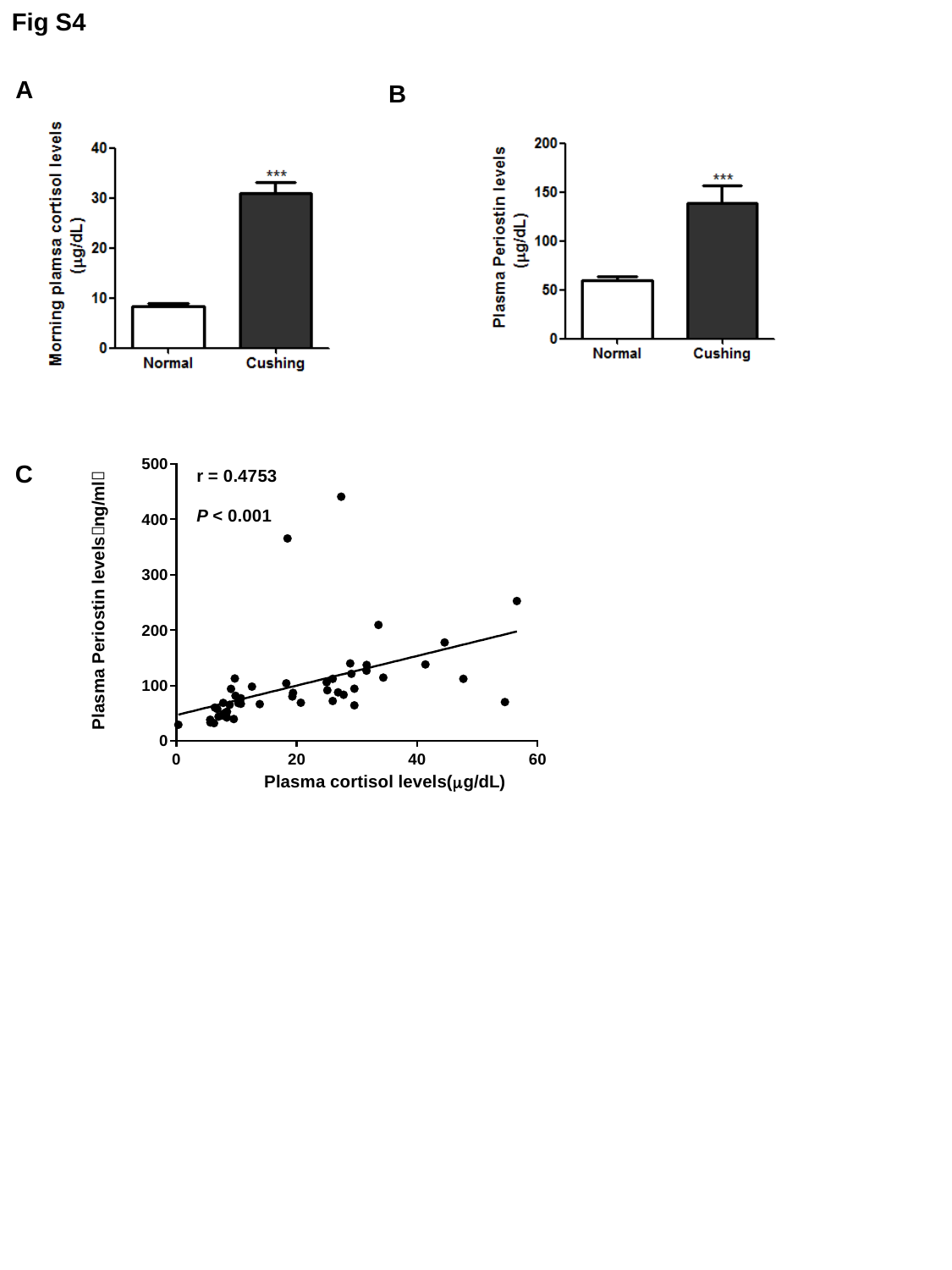

Fig S4
A
B
C

## Slide 5
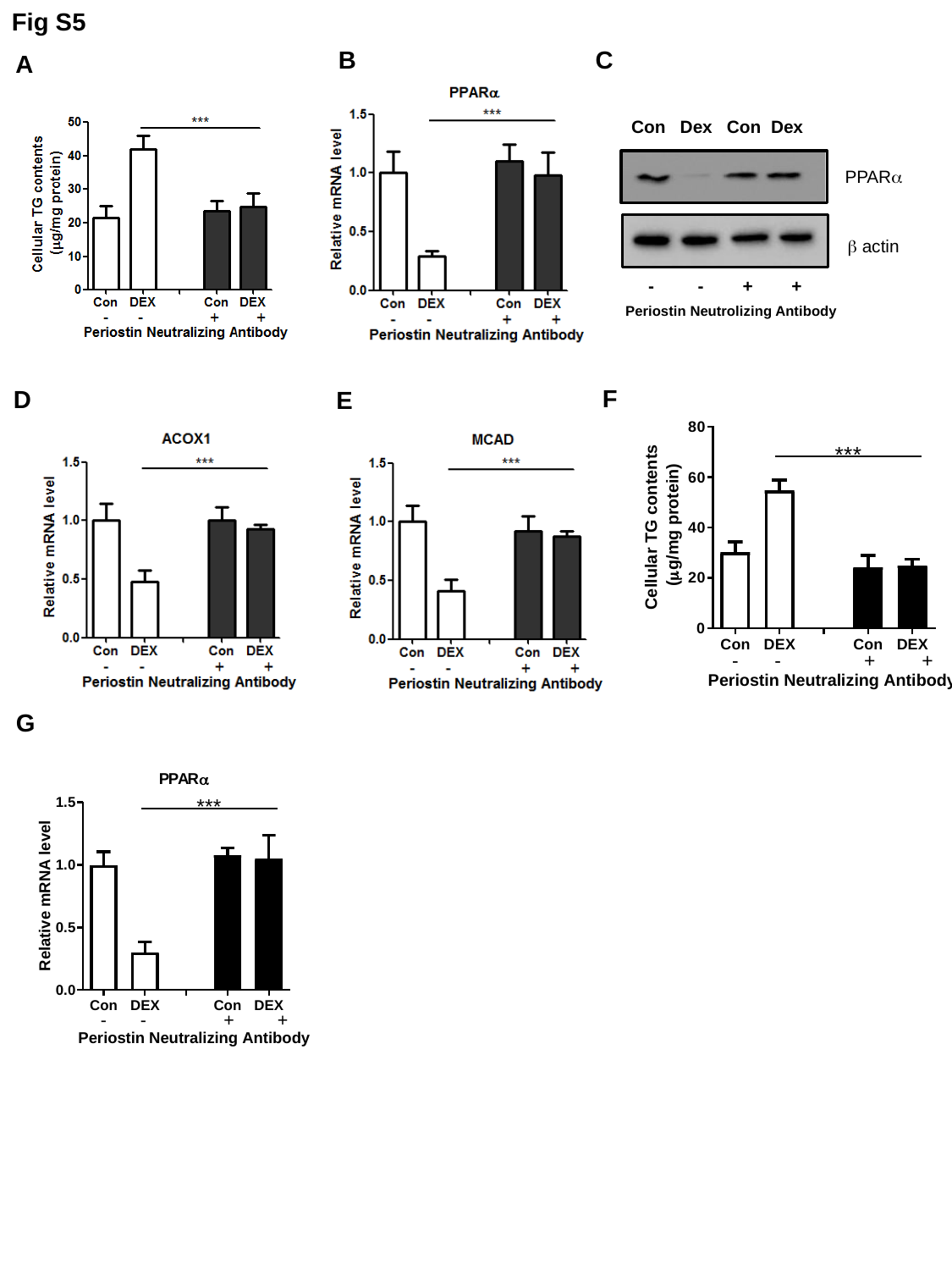

Fig S5
B
C
A
Con Dex Con Dex
PPARa
b actin
 - - + +
Periostin Neutrolizing Antibody
F
D
E
G

## Slide 6
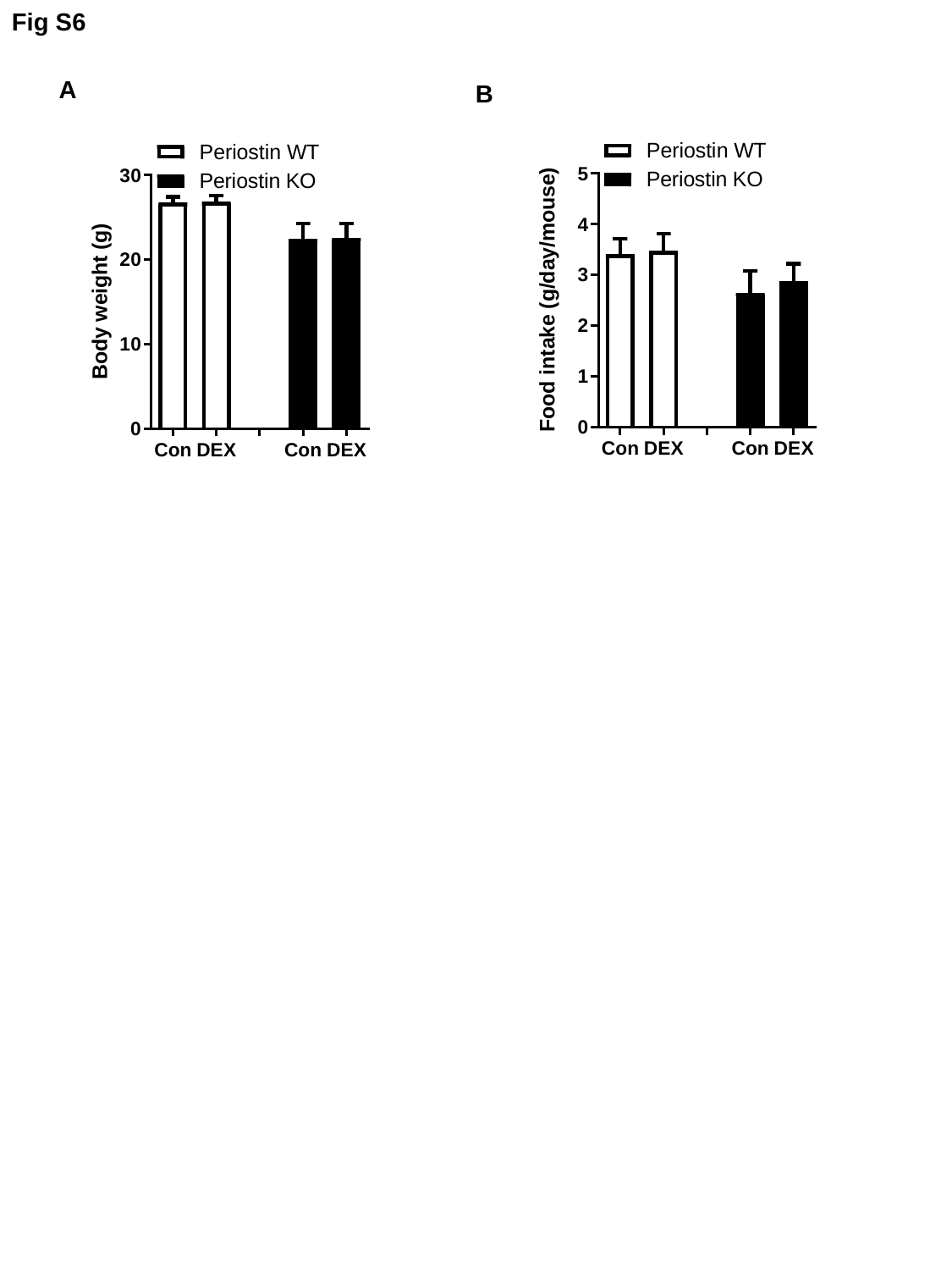

Fig S6
A
B
